# Assessment of genome-wide genetic variability and population divergence of four native Turkish sheep breeds

**DOI:** 10.1007/s00335-026-10213-8

**Published:** 2026-04-06

**Authors:** Bahar Argun Karslı, Eymen Demir, Umit Bilginer, Taki Karslı, Sarp Kaya, Huriye Doğru, Irmak Kara, Veli Atmaca, Duygu Kaşıkçı, Serdar Koçak, Serdar Yağcı, Murat Soner Balcıoğlu, Emiliano Lasagna, Fetih Gülyüz

**Affiliations:** 1 Department of Agricultural Biotechnology, Faculty of Agriculture, Eskişehir Osmangazi University, 26160 Eskişehir, Türkiye; 2 Department of Animal Science, Faculty of Agriculture, Akdeniz University, 07070 Antalya, Türkiye; 3 Department of Animal and Dairy Sciences, University of Wisconsin, Madison, WI 53706 USA; 4 Department of Animal Science, Faculty of Agriculture, Eskişehir Osmangazi University, 26160 Eskişehir, Türkiye; 5 Department of Medical Services and Techniques, Vocational School of Burdur Health Services, Burdur Mehmet Akif Ersoy University, 15100 Burdur, Türkiye; 6 Department of Animal Science, Faculty of Agriculture, Kırşehir Ahi Evran University, 40100 Kırşehir, Türkiye; 7 Department of Animal Science, Faculty of Agriculture, Isparta University of Applied Sciences, 32000 Isparta, Türkiye; 8 Department of Animal Science, Faculty of Veterinary Medicine, Afyon Kocatepe University, 03200 Afyonkarahisar, Türkiye; 9 Ministry of Agriculture and Forestry, General Directorate of Agricultural Research and Policies, 06800 Çankaya, Ankara Türkiye; 10 Department of Agricultural, Food and Environmental Sciences, University of Perugia, 06121 Perugia, Italy

## Abstract

Genetic variability is crucial for enhancing economically important traits and for developing effective conservation strategies in livestock populations. In this study, genome-wide genetic variability and population divergence were evaluated in four native Turkish sheep breeds, known as Dağlıç (DGL), Sakız (SKZ), Pırlak (PRL), and Pırıt (PRT), using 366,544 single nucleotide polymorphisms (SNPs) generated by double-digest restriction-site associated DNA sequencing (ddRADseq). Across the four populations, the mean minor allele frequency (MAF) and nucleotide diversity (π) were estimated at 0.320 and 0.295, respectively. Observed heterozygosity (*H*_*O*_) ranged from 0.314 in the DGL breed to 0.323 in the SKZ breed. Expected heterozygosity (*H*_*E*_) values were consistently higher than the observed values across all breeds, with an overall mean of 0.327. The inbreeding coefficient (*F*_*IS*_) was negative in all populations, varying from − 0.034 in PRL to − 0.019 in DGL. Population structure analyses based on discriminant analysis of principal components (DAPC), ADMIXTURE, and TreeMix revealed a high level of admixture between the PRL and PRT populations, whereas the DGL breed was genetically distinct. In addition, TreeMix inferred a migration edge from the SKZ breed to the PRL–PRT clade. The high level of genetic variability observed in native Anatolian sheep breeds should be conserved to support diverse breeding strategies and to mitigate potential future challenges, particularly genetic bottlenecks. Further studies employing improved sampling strategies are recommended to obtain more robust insights into the genetic structure of native Anatolian sheep, especially for the PRL and PRT populations.

**Supplementary Information:**

The online version contains supplementary material available at 10.1007/s00335-026-10213-8.

## Introduction

Türkiye is among the leading countries contributing to global animal genetic resources. The country hosts nearly 50 sheep breeds, including 33 native genotypes. The continous declines in their effective population sizes, on the other hand, remain one of the most pressing concerns among researchers (Kandemir and Taşkın 2022; Aydin et al. 2024; Yıldırır et al. 2025). Sheep breeding in Türkiye is predominantly carried out by smallholder farmers under extensive production systems, relying largely on native breeds adapted to diverse rural environments (Ameur Ameur et al. 2020; Koçak et al. 2024). Among native sheep breeds, Dağlıç (DGL), characterized by its fat-tail phenotype, has been reared in Anatolia for centuries and has consequently developed strong adaptation to environmental challenges. Owing to its superior adaptability, DGL has played a key role in crossbreeding programs in Türkiye. For instance, the Ramlıç breed was developed through crossbreeding between DGL (30–35%) and Rambouillet (65–70%) (Atav and Buğdaycı 2022). Similarly, the Acıpayam genotype originated from crossbreeding involving İvesi (50%), Ost Fries (25%), and DGL (25%) (Özbaşer and Akçapınar 2011). Originating from the Chios Islands, the Sakız (SKZ) breed is reared mainly along the Aegean coastal region. It exhibits distinctive phenotypic characteristics, including a thin, fatless tail and black-and-white markings on the face and legs (Bayraktar 2024). SKZ is recognized as one of the most prolific sheep breeds, with litter sizes reaching up to 2.3 and relatively high lamb survival rates (76.6–88.8%) (Ceyhan et al. 2009). According to the literature, the Pırlak (PRL) sheep originated from crossbreeding between DGL and Kıvırcık sheep (Çelikeloğlu et al. 2018). However, the scarcity of available knowledge makes it difficult to determine whether the breed was developed locally by sheep breeders or emerged through crossbreeding practice (Öner et al. 2014). PRL sheep are typically hornless, with a white coat color and black spots around the facial area and ears (Kuru et al. 2020). The PRL breed is also referred to as Pırıt (PRT) by farmers (Çelikeloğlu and Tekerli 2014; Taşkın and Kandemir 2022). However, a recent microsatellite-based study reported that these populations are genetically distinct (Ata et al. 2025).

The use of native sheep breeds in animal breeding entails both advantages and limitations. The main obstacle to native sheep breeds stems from their low or moderate productivity. Indeed, due to a lack of long-term selective breeding programs, native Turkish sheep breeds have not been specialized for any economically important traits: This fact leads to their categorization as multipurpose breeds that produce meat, milk, and wool in moderate levels. However, they have developed adaptations against diverse environmental challenges, ranging from climatic conditions to disease resistance (Soysal et al. 2005; Karsli et al. 2020; Bayraktar 2024). In addition, native sheep breeds are well suited to grassland-based production systems. This kind of system not only allows efficient utilization of natural pastures and rangelands but also enables low-cost animal production in rural areas (Bozkurt et al. 2023; Yurdagül et al. 2023). Beyond their economic value, native Turkish sheep breeds represent an important genetic resource for genomic studies investigating genome-wide variability. These populations have been raised in close geographic proximity to the center of sheep domestication. Therefore, they are expected to retain ancient genetic variants. Indeed, sheep populations located near the domestication center are hypothesized to harbor higher levels of genetic diversity, which is predicted to decline gradually with increasing geographic distance (Kijas et al. 2009; Yıldırır et al. 2025).

Genetic variability causes differences among animals regarding morphological, behavioral, and adaptive traits (Demir 2024). Improvement of economically important traits (meat and milk in particular) in breeding studies could only be achieved in the presence of adequate genetic variability. In addition, the level of genetic variability allows for giving priority to a specific population in conservation programs. Therefore, monitoring the genetic variability within and between native sheep breeds is essential for farmers to improve breeding strategies and for authorities to develop comprehensive conservation programs (Olschewsky and Hinrichs 2021). Recent advances in molecular genotyping technologies have enabled the assessment of genetic variability across scales ranging from single loci to the whole genome. Among contemporary approaches, double-digest restriction-site associated DNA sequencing (ddRADseq) is based on the enzymatic digestion of genomic DNA using two restriction enzymes. This technique allows the recovery of thousands of genome-wide single nucleotide polymorphisms (SNPs) (Peterson et al. 2012). Compared with array-based technologies, ddRADseq substantially reduces genotyping costs while simultaneously decreasing genome complexity. Owing to its advantages, including cost efficiency, accuracy, and the absence of a requirement for prior genomic information, ddRADseq has been widely applied in some of the farm animals, such as cattle (Demir et al. 2023; Nayak et al. 2025), buffalo (Tyagi et al. 2021), pig (Vani et al. 2024), sheep (Argun Karslı et al. 2024), goat (Bilginer et al. 2025), and chicken (Doublet et al. 2024), for a broad range of molecular genetic analyses.

Genome-wide molecular genetic studies aimed at characterizing genetic variability in native Turkish sheep breeds remain limited (Bayraktar 2024; Demir 2024; Karsli 2024, 2025; Karabaş and Yılmaz 2025). However, more comprehensive analyses are required to gain deeper insight into the genetic architecture of the PRL and PRT populations. Besides, the contribution of other sheep breeds, such as DGL and SKZ, for shaping the genetic architecture of PRL and PRT breeds should be addressed via advanced molecular genotyping techniques. Accordingly, this study was designed to assess genetic variability and population divergence of four native Turkish sheep (DGL, SKZ, PRL, and PRT) via genome-wide genetic data obtained from the ddRADseq technique.

## Materials and methods

### Sampling strategy and DNA extraction

In this study, at least three independent herds were visited for each sheep breed, and a total of 80 unrelated animals (20 individuals per population) were sampled from the DGL, SKZ, PRL, and PRT breeds. The sampled animals were between 2 and 3 years of age, and each population consisted of 17 females and 3 males. Samples from the DGL and PRT populations were collected from Afyon and Isparta provinces, respectively, whereas PRL and SKZ samples were obtained from Antalya province. Breed-specific characteristics, including morphological traits and available pedigree information, were used to identify and sample individuals from the DGL and SKZ breeds. DGL breed was registered in the Community-based animal improvement project (TAGEM03DAG2011-01) operated by the General Directorate of the Agricultural Research and Policies of the Ministry of Agriculture and Forestry of Türkiye. In contrast, due to the absence of officially defined distinguishing traits between the PRL and PRT populations, individuals from these breeds were selected based on information provided by local breeders. For each animal, 5 mL of whole blood was drained from the jugular vein by qualified veterinarians to minimize physical stress. Collected blood samples were subjected to DNA extraction using the GeneJET Genomic DNA Purification Kit (Thermo Scientific, K0721). DNA integrity was assessed by agarose gel electrophoresis, and DNA concentration was quantified using a Qubit 4™ fluorometer (Thermo Fisher Scientific) with the double-stranded DNA High Sensitivity (HS) assay kit (Invitrogen).

### Genomic library preparation and sequencing

In this study, a pipeline explained by Peterson et al. (2012) was used to prepare genomic libraries. In brief, total DNA was digested with two restriction enzymes, known as *EcoR*I and *Msp*I, followed by the ligation of P1 adapters. All DNA fragments were pooled, sheared, and P2 adapters were fused by T4 DNA ligase. Further, DNA fragments with 300–500 bp length were selected and enriched by the PCR technique to obtain clean DNA libraries ready for sequencing. DNA libraries were sequenced by the Illumina NovaSeq 6000 instrument with the paired-end option (2 × 150 bp) to obtain short reads.

### Variant calling and filtering

Variant calling and filtering were performed following the pipeline described in a recent ddRADseq-based study on native sheep breeds (Argun Karsli et al. 2024). In this regard, the *process_radtags* command of the Stacks 2 program (Rochette et al. 2019) was utilized to assign each read to individuals. Default parameters of the fastp program (Chen et al. 2018) were used to filter assigned reads. Cleaned reads were further aligned to the reference genome of *Ovis aries* (ARS-UI_Ramb_v3.0) via default parameters of the Bowtie2 algorithm (Langmead and Salzberg 2012). Biallelic SNPs located on autosomes with high read depth (20 ≤ D ≤ 100) and quality (Q ≥ 20) were retrieved via the BCFtools pipeline (Danecek et al. 2021). The standard PLINK 1.9 (Chang et al. 2015) filtration flags (--maf 0.05, --geno 0.1, and --mind 0.1) were applied to obtain the final dataset.

## Bioinformatics analyses

### Genomic verification of pedigree records

In this study, we relied on pedigree records and/or oral interviews with breeders to sample unrelated animals, which could be verified via genomic relatedness values. Therefore, genomic relatedness among sampled individuals was evaluated using the identity-by-descent (IBD) approach implemented in PLINK 1.9 (Chang et al. 2015), while the results were visualised as a genomic kinship heatmap by using the pheatmap package (Kolde and Kolde 2015).

### Distribution of runs of homozygosity and inbreeding

Runs of homozygosity (ROH) islands were identified to assess genome-wide homozygosity and inbreeding across four native Turkish sheep breeds. A consecutive runs approach implemented in the detectRUNS package (Biscarini et al. 2019) with pre-defined parameters (minimum number of consecutive SNPs for each ROH > 50, minimum length of a ROH: 50, maximum gap between consecutive homozygous SNPs: 1 Mb, maximum number of SNPs with missing genotypes: 2, and maximum number of heterozygous SNPs in a ROH: 1) adopted for native Turkish sheep breeds by a recent study (Argun Karsli et al. 2024) was utilized to identify ROH islands. Each ROH island, calculated per individual, was categorised into 0 to < 2 Mb, 2 to < 4 Mb, 4 to < 8 Mb, 8 to < 16 Mb, and ≥ 16 Mb clusters based on their physical lengths. The inbreeding coefficient based on ROH (*F*_*ROH*_) is calculated as the sum of the length of all ROH per individual as a proportion of the total autosomal SNP coverage. Using this methodology, F_ROH_ was estimated for each ROH category.

Genome-wide heterozygosities, including observed (*H*_*O*_) and expected (*H*_*E*_), and inbreeding coefficients (*F*_*IS*_) across four Anatolian sheep, were estimated by the HierFstat program (Goudet 2005). Minor allele frequency (MAF) and nucleotide diversity (π) were calculated via SNPRelate (Zheng et al. 2012) and VCFtools (Danecek et al. 2011) programs, respectively. The historical trends in effective population size (*Ne*) up to 400 generations ago were estimated using a linkage disequilibrium (LD)-based method implemented in SNeP v1.1 software (Barbato et al. 2015). The software estimates past *Ne* trajectories by exploiting the relationship between pairwise linkage disequilibrium (r²) and recombination rate across the genome. To provide complementary evidence for demographic inference and genomic structure, LD decay patterns were independently evaluated using PopLDdecay (Zhang et al. 2019). Pairwise r² values were calculated, and LD decay was assessed as a function of physical distance between SNPs. A graphical distribution of *Ne* values per generation and LD decay based on physical distance was visualised via the ggplot2 package (Wickham 2011). Genetic distance values among individuals and populations were calculated in the StAMPP package (Pembleton et al. 2013). These values were further processed by the MEGA12 program (Kumar et al. 2024) to construct Neighbor-Joining (NJ) trees at individual and breed levels. Discriminant analysis of principal components (DAPC) was carried out by using the adegenet package (Jombart 2008) to visualise the genetic structure of sheep breeds based on the separation of individuals among predefined groups. ADMIXTURE v1.3.0 (Alexander et al. 2009) was employed to evaluate the genetic admixture among individuals across different numbers of ancestral populations (K = 2 to 4). The results of ADMIXTURE analyses, such as the cross-validation values and the Q matrix for each K value, were further processed via ggplot2 (Wickham 2011) and the BITE package v.2 (Milanesi et al. 2017) for a better visualisation. Genetic relationships, such as splitting and mixing, among the four native Turkish sheep were further assessed via the TreeMix approach (Pickrell and Pritchard 2012). The analysis was initially performed without any migration events (m = 0) to infer the underlying population tree topology and to evaluate its consistency with NJ tree analysis. Subsequently, the program was run again with the options of 1.000 blocks of SNPs and 20 iterations for migration events ranging from m = 1 to m = 5. The likelihood values were visualised by using the ggplot2 package (Wickham 2011) to choose the optimal number of migration events (m). The TreeMix graphs corresponding to both the no-migration event (m = 0) and the optimal migration model were generated using the BITE package v2 (Milanesi et al. 2017).

## Results

### ddRADseq processing and SNP calling

In this study, 1.5 billion raw reads at 150 bp were detected via sequencing across four native Turkish sheep. A large percentage of short reads (95.04%) were successfully aligned to the sheep reference genome. After a strict filtration process, 72 samples across DGL (*n* = 18), SKZ (*n* = 19), PRL (*n* = 20), and PRT (*n* = 15) genotyped with 366.544 SNPs were recovered to conduct genetic variability and population structure analyses.

### Genomic verification of pedigree records

In this study, the IDB approach was utilized to genetically verify pedigree records and the breeder’s oral information regarding relatedness status among sampled animals. A heatmap constructed based on pairwise genomic kinship values among individuals is given in Supplementary Fig. S1. Most pairwise comparisons showed the values below 0.15, indicating the absence of close kinship among individuals. However, one pair of individuals (SKZ10 and SKZ15) exhibited a kinship value of 0.421, consistent with first-degree relatedness. This finding confirms that pedigree records and breeders’ information were consistent with estimated genomic relatedness values.

### Distribution of runs of homozygosity and inbreeding

In this study, a total of 1501, 988, 886, and 969 ROH islands were identified in DGL, PRL, PRT, and SKZ sheep breeds, respectively. A significant ratio of these ROH islands (99%) was detected for 0–2 Mb cluster for all breeds, while no ROH segments longer than 4 Mb were observed. The mean values for number and length of ROH segments, as well as ROH-based inbreeding values per two different categories (0–2 and 2–4 Mb) are summarised in Table 2.

**Table 1:** An overview of ROH characterisation and ROH-based inbreeding across four native Turkish sheep breeds

ROH category	0–2 Mb			2–4 Mb		
Parameter	ROH number (Mean ± SD)	ROH length (Mean ± SD)	F_ROH_ (Mean ± SD)	ROH number (Mean ± SD)	ROH length (Mean ± SD)	F_ROH_ (Mean ± SD)
DGL	83.388 ± 39.760	63.750 ± 32.640	0.002 ± 0.001	2.000 ± 1.600	4.460 ± 3.540	0.001 ± 0.001
PRL	49.400 ± 45.640	35.990 ± 36.360	0.001 ± 0.001	1.500 ± 0.700	3.940 ± 1.730	0.001 ± 0.000
PRT	59.066 ± 63.880	44.600 ± 50.960	0.001 ± 0.002	1.000 ± 0.000	2.540 ± 0.260	0.001 ± 0.000
SKZ	51.000 ± 45.630	38.400 ± 36.710	0.001 ± 0.001	1.000 ± 0.000	2.630 ± 0.170	0.001 ± 0.000

Among the populations, DGL exhibited the highest mean number of short ROH segments (83.388), followed by PRT (59.066), SKZ (51.000), and PRL (49.400) according to 0–2 Mb category (Table 2). Similarly, the lowest and highest mean total length of short ROH segments (0–2 Mb) were observed in PRL (35.990 Mb) and DGL (63.750 Mb) breeds, respectively (Table 2). Genome-wide inbreeding coefficients based on ROH were generally low across all populations. For short ROH segments (0–2 Mb), the highest mean *F*_*ROH*_ was observed in DGL (0.002), whereas this value was calculated as 0.001 for the remaining sheep breeds (Table 2). The contribution of 2–4 Mb segments to genomic inbreeding was minimal in all populations (0.001).

### Genetic variability and population divergence

Genome-wide genetic variability parameters and inbreeding coefficients per studied sheep populations are given in Table 2.

**Table 2:** Genetic variability parameter across four native Turkish sheep

Breed	MAF	π	H_O_	H_E_	F_IS_
DGL	0.310	0.280	0.314	0.320	−0.019
SKZ	0.319	0.290	0.323	0.327	−0.021
PRL	0.332	0.300	0.321	0.333	−0.034
PRT	0.320	0.300	0.320	0.330	−0.030
Mean	0.320	0.295	0.319	0.327	−0.026

DGL: Dağlıç; SKZ: Sakız; PRL: Pırlak; PRT: Pırıt; MAF: Minor allele frequency; π: Nucleotide diversity; *H*_*O*_: Observed heterozygosity; *H*_*E*_: Expected heterozygosity; *F*_*IS*_: Inbreeding coefficient.

Across all populations, the mean MAF and nucleotide diversity were estimated at 0.320 and 0.295, respectively (Table 2). Observed heterozygosity, a key indicator of genetic variability, ranged from 0.314 in the DGL breed to 0.323 in the SKZ breed, with an overall mean of 0.319 across populations. Expected heterozygosity values were consistently higher than the corresponding observed values in all four native sheep breeds (Table 2). Negative inbreeding coefficient values were detected in all populations, with a mean of − 0.026, indicating no evidence of inbreeding.

**Fig. 1:**
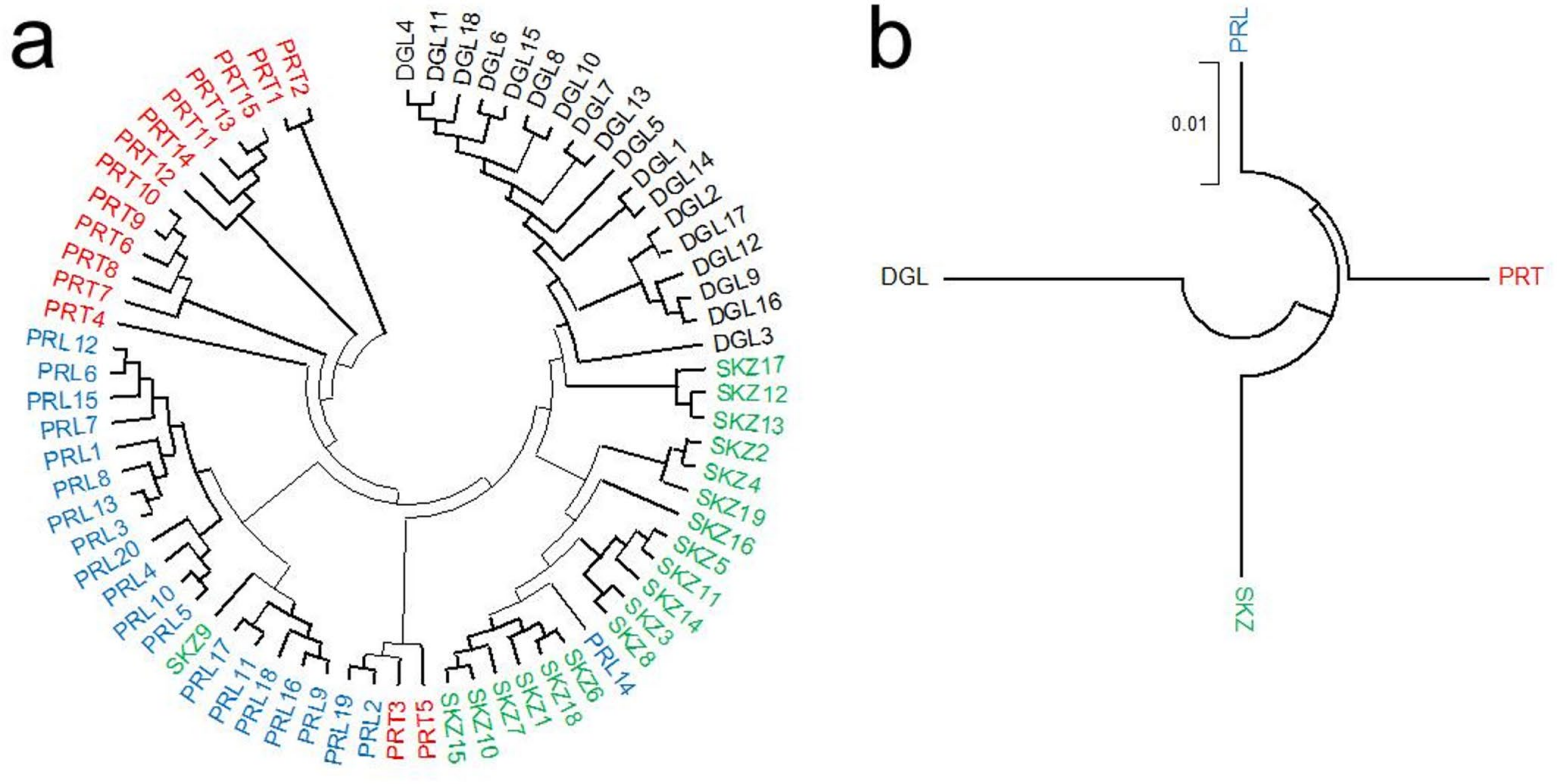
Genetic distance-based NJ trees per **a** individual and **b** population level

The phylogenetic tree based on genetic distance estimates indicated that the DGL and SKZ breeds formed distinct clusters at both the individual (Fig. 1a) and breed (Fig. 1b) levels, whereas the PRL and PRT populations clustered within the same phylogenetic clade. At the individual level, one SKZ individual (SKZ9) was grouped with the PRL–PRT clade, while one PRL individual (PRL14) was assigned to the SKZ cluster. The lowest genetic distance (0.020) was observed between the PRL and PRT populations, whereas the highest genetic distance (0.039) was detected between the DGL and PRL populations.

**Fig. 2:**
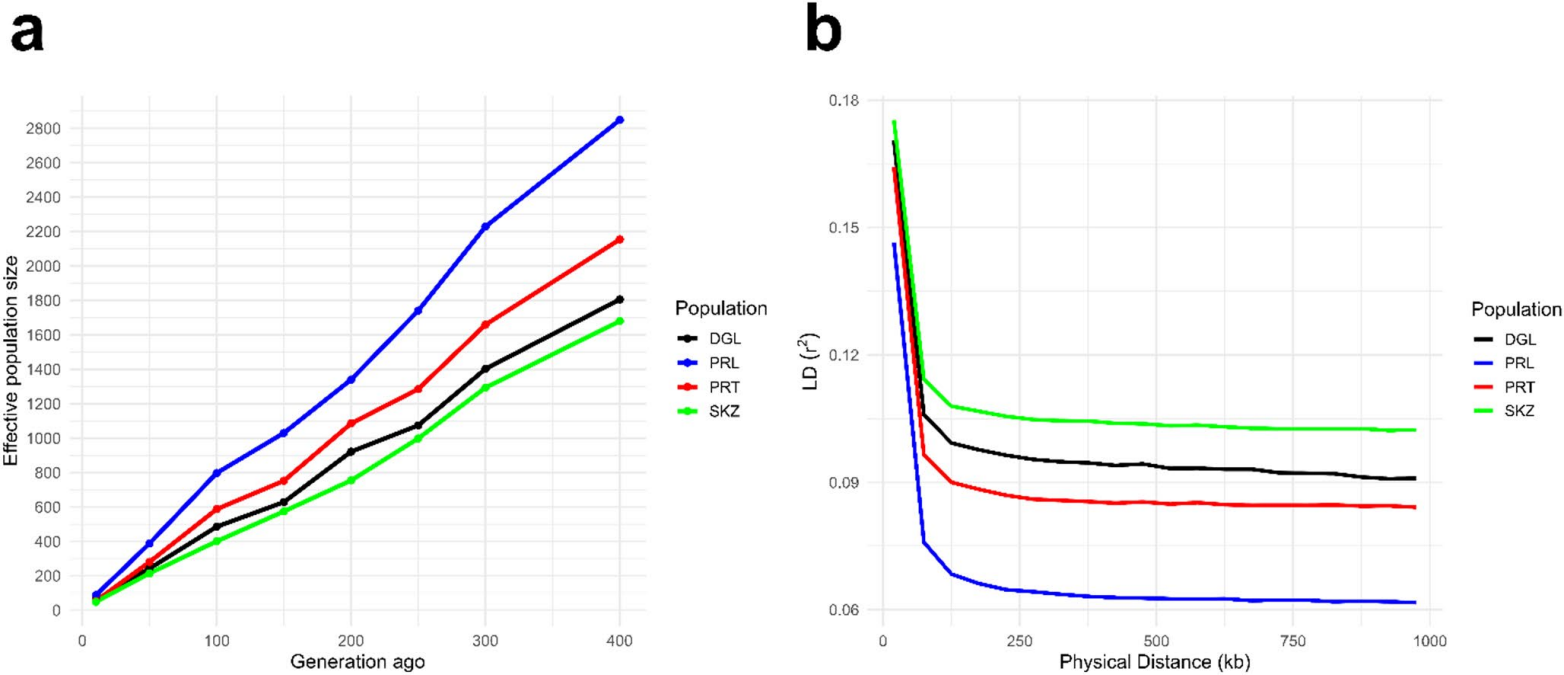
Trend in **a** *Ne* values across generations and **b** LD decay based on different physical distance values

The reconstructed historical effective population size trajectories are illustrated in Fig. 2a. Among the studied breeds, the highest *Ne* estimates were observed in the PRL population across the evaluated time scale. Approximately 400 generations ago, *Ne* in PRL approached 2800 individuals, indicating a comparatively large ancestral population size. In contrast, SKZ exhibited lower historical Ne values, estimated at around 1600 individuals at the same time depth. PRT followed PRL, whereas DGL and SKZ showed relatively smaller effective population sizes throughout the examined generations. Consistent with these demographic patterns, the LD decay analysis (Fig. 2b) revealed the most pronounced decline in linkage disequilibrium with increasing physical distance in the PRL population. The steeper LD decay in PRL supports its larger effective population size, as higher Ne is expected to accelerate the breakdown of LD over generations. Conversely, breeds with lower *Ne* values exhibited a slower LD decay pattern, reflecting stronger persistence of linkage disequilibrium across the genome.

**Fig. 3:**
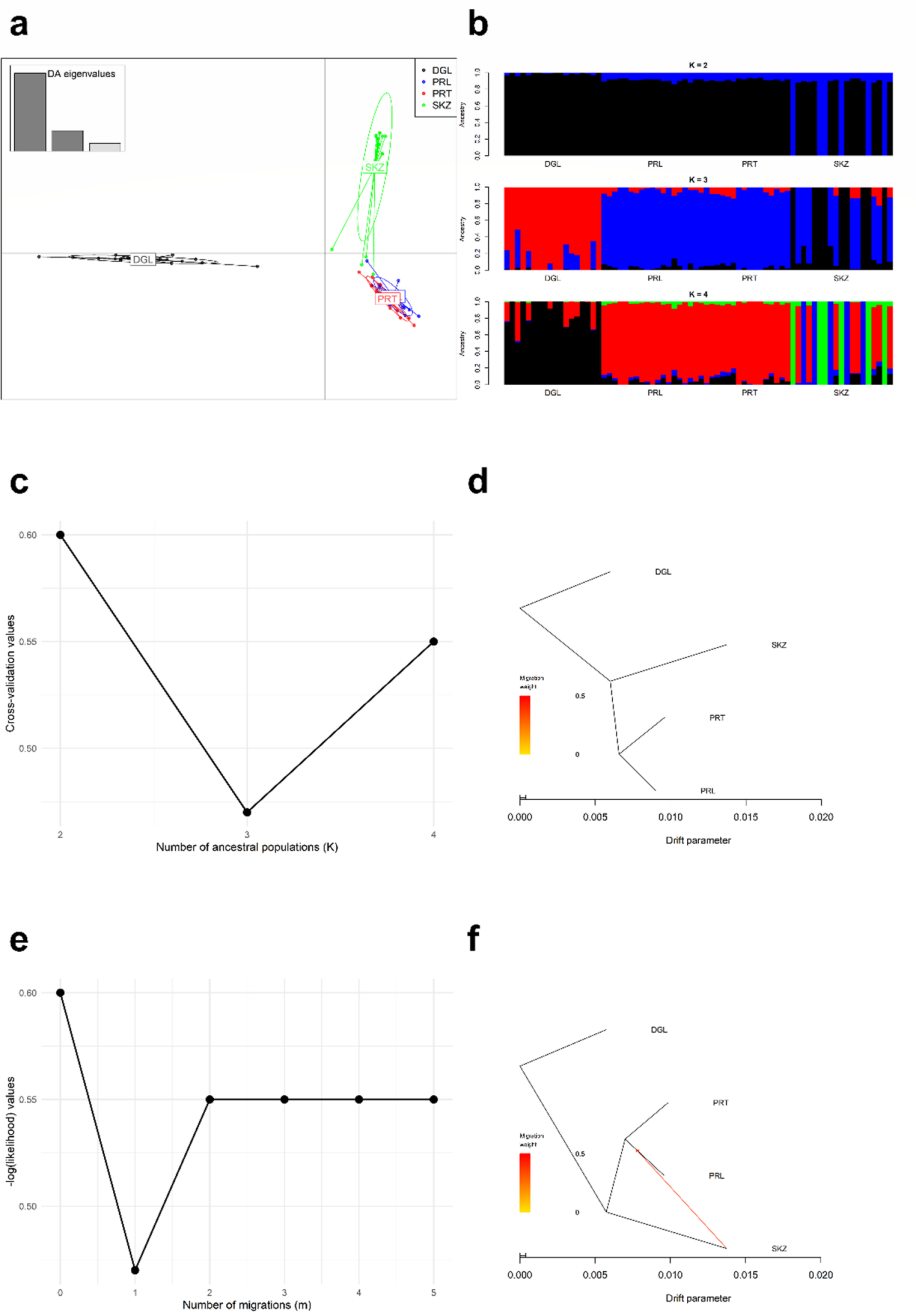
A graphical representation of **a** DAPC analysis, **b** ADMIXTURE analysis, **c** the distribution of cross-validation errors across different K values, **d** Treemix analysis without migration events, **e** the distribution of likelihood values for different migration events, and **f** Treemix analysis with a migration event

DAPC analysis showed consistency with the findings of the phylogenetic tree, in which PRL and PRT were admixed close to some samples from the SKZ breed, while DGL was genetically distinct (Fig. 3a). In ADMIXTURE analysis, PRL and PRT were assigned to a similar ancestral population (Fig. 3b). The cross-validation values (Fig. 3c) confirmed that the optimal number of ancestral populations was 3, in which DGL was genetically distinct, while an admixture from PRT-PRL to SKZ was identified (Fig. 3b). TreeMix algorithm without migration was drawn to screen population splits, in which PRL and PRT were still clustered together, while DGL was genetically different (Fig. 3d). The likelihood values verified that the optimal number of migration events was 1 (Fig. 3e), in which a migration edge was drawn from SKZ to the PRL-PRT clade (Fig. 3f).

## Discussion

### Genetic variability and effective population size

Genetic variability represents a key indicator for sheep breeders in designing and improving breeding strategies, as well as for decision-makers in prioritizing conservation programs. Accordingly, the genetic variability of native Turkish sheep breeds has been investigated for several decades using a variety of molecular genotyping approaches. Early studies predominantly relied on microsatellite (Gutiérrez-Gil et al. 2006; Öner et al. 2014; Yılmaz et al. 2014, 2015; Ameur Ameur et al. 2020; Karslı et al. 2020; Kırıkçı et al. 2020; Ozmen et al. 2020; Alarslan et al. 2021; Ata et al. 2025; Yıldırır et al. 2025) and mitochondrial DNA (mtDNA) markers (Demirci et al. 2013; Oner et al. 2013; Kirıkçı et al. 2018; Yağcı et al. 2020) to assess genetic diversity in native Turkish sheep populations. However, these marker systems are limited in number and do not adequately represent genome-wide variation. These limitations have driven the application of high-density SNP array technologies (Bayraktar 2024; Karabaş and Yılmaz 2025) and next-generation sequencing (NGS)-based approaches (Demir 2024; Karslı 2024, 2025) to investigate genome-wide genetic variability in native Turkish sheep breeds. In this context, the present study provides an important contribution by assessing genetic variability in four Anatolian sheep populations using 366,544 genome-wide SNPs, a substantially higher marker density than that reported in most previous studies (Bayraktar 2024; Demir 2024; Karslı 2024; Karabaş and Yılmaz 2025). Indeed, earlier studies on native Turkish sheep have reported SNP counts ranging from 46,314 (Bayraktar 2024) to 351,539 (Karslı 2024). The genetic variability parameters estimated in the present study are largely consistent with values reported for other native Turkish sheep breeds. Specifically, the mean MAF and nucleotide diversity were estimated at 0.320 and 0.295, respectively. A comparable MAF value (0.320) was reported for Kangal Akkaraman sheep based on 238,103 SNPs identified using genotyping-by-sequencing (Demir 2024). Similarly, MAF and nucleotide diversity values of 0.340 and 0.235, respectively, were reported for Anatolian Merino sheep (Karslı 2024). Moreover, a mean MAF of 0.314 was reported for Akkaraman, Güneykaraman, Karakaş, and Morkaraman breeds based on 296,097 SNPs obtained via ddRADseq (Karslı 2024). In contrast, a lower MAF value (0.300) was observed in Eşme sheep genotyped using the OvineSNP50K array (Karabaş and Yılmaz 2025). Observed heterozygosity values in the present study ranged from 0.314 in the DGL breed to 0.323 in the SKZ breed, aligning well with previously reported estimates, which ranged from 0.258 in Anatolian Merino sheep (Karslı 2024) to 0.388 in Eşme sheep (Karabaş and Yılmaz 2025).

A review of the literature indicates that the genetic variability parameters estimated for the DGL, SKZ, PRL, and PRT breeds are generally higher than those reported for sheep populations genotyped using NGS approaches in other regions, including China (Cheng et al. 2020; Shi et al. 2023), Sudan (Amane et al. 2022), and Latvia (Gudra et al. 2024). The relatively high levels of genetic variability observed in native Turkish sheep breeds are not unexpected, given that Türkiye is located within the Fertile Crescent, which is recognized as one of the primary centers of sheep domestication. Compared to DGL and SKZ, the detection of a slightly higher variability in PRL and PRT populations is not surprising since they are crossbreeds.

In the present study, the predominance of short ROH segments (0–2 Mb) and the absence of long ROH (> 4 Mb) indicate that recent inbreeding is limited across all studied populations. Instead, the observed autozygosity appears to reflect historical demographic processes such as genetic drift or long-term population structure. Among the populations, DGL displayed comparatively higher ROH number, total ROH length, and F_ROH_ value at 0–2 Mb class, suggesting a relatively higher level of background autozygosity. However, the overall F_ROH_ values remained low (< 0.003), indicating that none of the populations are currently experiencing severe inbreeding. Similarly, negative inbreeding coefficient values were detected across all analyzed sheep breeds, indicating an absence of inbreeding. Comparable negative *F*_*IS*_ values have been reported for several other native Anatolian sheep breeds, including Karakaş, Norduz, Akkaraman, Güneykaraman, and Morkaraman (Bayraktar 2024; Karslı 2024). In contrast, positive *F*_*IS*_ estimates have also been documented in certain Turkish sheep populations, ranging from 0.01 in Kangal Akkaraman (Demir 2024) to 0.029 in the Eşme breed (Karabaş and Yılmaz 2025). Absence of inbreeding is promising for the sustainable use of the present native sheep breeds in the future.

In the present study, a declining trend in *Ne* was observed across the DGL, SKZ, PRL, and PRT populations, with the highest *Ne* values consistently detected in the PRL breed under all evaluated scenarios. This observation may be attributed to the fact that the PRL and PRT sheep were originally obtained through crossbreeding practices and subsequently bred within their own populations. Similar downward trends in *Ne* have previously been reported for Anatolian Merino and Kangal Akkaraman sheep (Demir 2024; Karslı 2024), as well as for several native sheep breeds in Latvia (Gudra et al. 2024) and China (Cheng et al. 2020). A potential limitation of the present study is the relatively small sample size for each population, which may affect the precision of some genetic parameter estimates. Future studies with larger and more geographically diverse samples are recommended to validate and refine the observed genetic patterns.

### Population structure and divergence

Considering previous molecular studies carried out on native Turkish sheep, the genetic origins of the PRL and PRT breeds remain insufficiently characterized among the studied populations. Consequently, genome-wide genetic data, rather than microsatellite or mtDNA markers, are required to gain deeper insight into this knowledge gap. High-density genome-wide data generated using SNP arrays or NGS platforms are particularly effective in detecting genetic divergence among breeds and in elucidating population structure, especially for populations with unclear or complex genetic backgrounds. Moreover, even when sheep breeds share a common ancestral origin, they may diverge genetically over time as a result of geographic isolation and human-mediated breeding practices. Indeed, farmer activities, such as transporting animals from one geographic zone to another, may lead to genetic admixture among different sheep breeds (Ozmen et al. 2020). Therefore, population structure results should be interpreted with caution, taking into account both historical demographic processes and recent anthropogenic influences.

A recent and up-to-date study based on 18 microsatellite loci implied that PRL were genetically close to Kıvırcık, while PRT were genetically distinct (Ata et al. 2025). In this study, however, genetic structure algorithms implied that the PRL and PRT were admixed. These findings may seem inconsistent, but in this study, the sampling sites of the PRL (Antalya province) and PRT (Isparta province) populations are very close. A human-mediated animal transportation between Antalya and Isparta may lead to a high level of genetic admixture between the PRL and PRT. Moreover, although there is no documented evidence in the literature, breeders claim that the PRT populations raised in Isparta are phenotypically similar to the PRL population raised in Antalya. In contrast, the PRL populations raised in Afyon are considered to be distinct. Accordingly, future studies adopting more comprehensive sampling strategies, particularly including geographically distant populations, are required to reconcile these contrasting results and to better resolve the genetic origins of the PRL and PRT breeds.

Furthermore, a migration event from the SKZ population toward the PRL–PRT clade was identified in the present study. To the best of our knowledge, no previous studies have explicitly evaluated migration events between SKZ and other native Turkish sheep breeds. Therefore, additional genome-wide studies are needed to validate this inferred migration and to clarify historical gene flow patterns among native Anatolian sheep populations.

Another finding of the current study was that the DGL breed was genetically distinct from other breeds. This result is consistent with past breeding practices of the DGL breed. Indeed, several studies have mentioned that DGL has been used to obtain novel sheep breeds or types (Ramlıç and Acıpayam) (Özbaşer and Akçapınar 2011; Atav and Buğdaycı 2022), no literature showing genetic introgression with SKZ, PRL, and PRT is available. Such breeding management practices likely contributed to the maintenance of the genetic distinctiveness of DGL. Supporting this observation, Özdemir et al. (2011) examined genetic distances among nine native Turkish sheep breeds using polymorphic blood protein markers and reported that DGL formed a genetically distinct cluster in phylogenetic analyses, clearly separated from major Anatolian sheep breeds such as Akkaraman, Morkaraman, İvesi, and Kıvırcık.

## Conclusion

This study evaluated genome-wide genetic variability and population divergence in four native Turkish sheep populations, namely DGL, SKZ, PRL, and PRT, using 366,544 SNPs generated through the ddRADseq approach. The findings confirm that native sheep breeds harbor high levels of genome-wide genetic variability. The conservation of this variability is crucial not only for enabling farmers to implement diverse and effective breeding programs but also for maintaining the adaptive capacity of these populations to cope with a wide range of environmental challenges.

Despite the high genetic diversity observed, a declining trend in effective population size was detected across the studied breeds, underscoring the need for periodic monitoring using modern molecular genotyping techniques to mitigate the potential adverse effects of genetic bottlenecks. Population structure analyses, including DAPC and phylogenetic reconstruction, consistently revealed a high degree of genetic overlap between PRL and PRT populations, with no distinct clustering separating these groups. This pronounced genomic similarity indicates substantial shared ancestry or ongoing gene flow between PRL and PRT via human-mediated animal movement, at least within the sampled individuals. Accordingly, our genome-wide data do not provide compelling evidence for clear genetic differentiation between these populations. While PRL and PRT are traditionally recognized as separate breeds based on phenotypic traits and local breeding practices, the present findings suggest that their genetic distinction may be limited within the current sampling framework. Therefore, the taxonomic separation of PRL and PRT should be interpreted with caution. In this context, further studies employing more comprehensive and geographically diverse sampling strategies are required to draw definitive conclusions regarding the genetic structure and conservation status of the PRL and PRT populations. Besides, investigating not only genetic diversity and population structure but also selection signatures and genomic regions associated with production and adaptive traits through genome-wide association studies has the potential to provide deeper insight into their genomes. Such approaches will help to identify differentiated regions across the genome and will also contribute significantly to the development of effective conservation and breeding strategies.

## Supplementary Information

Below is the link to the electronic supplementary material.

Supplementary material 1 (DOCX 3246.9 kb)

## Data Availability

The data that support the findings of this study are available from the corresponding author upon reasonable request.
