# Supplementary material for: Assessment of genome-wide genetic variability and population divergence of four native Turkish sheep breeds
Source: Mamm Genome. 2026 Apr 6;37(1):51. doi: 10.1007/s00335-026-10213-8 (PMC13053530; doi:10.1007/s00335-026-10213-8)
Supplement: Supplementary file 1 — Supplementary material 1 (DOCX 3246.9 kb) [file 335_2026_10213_MOESM1_ESM.docx]

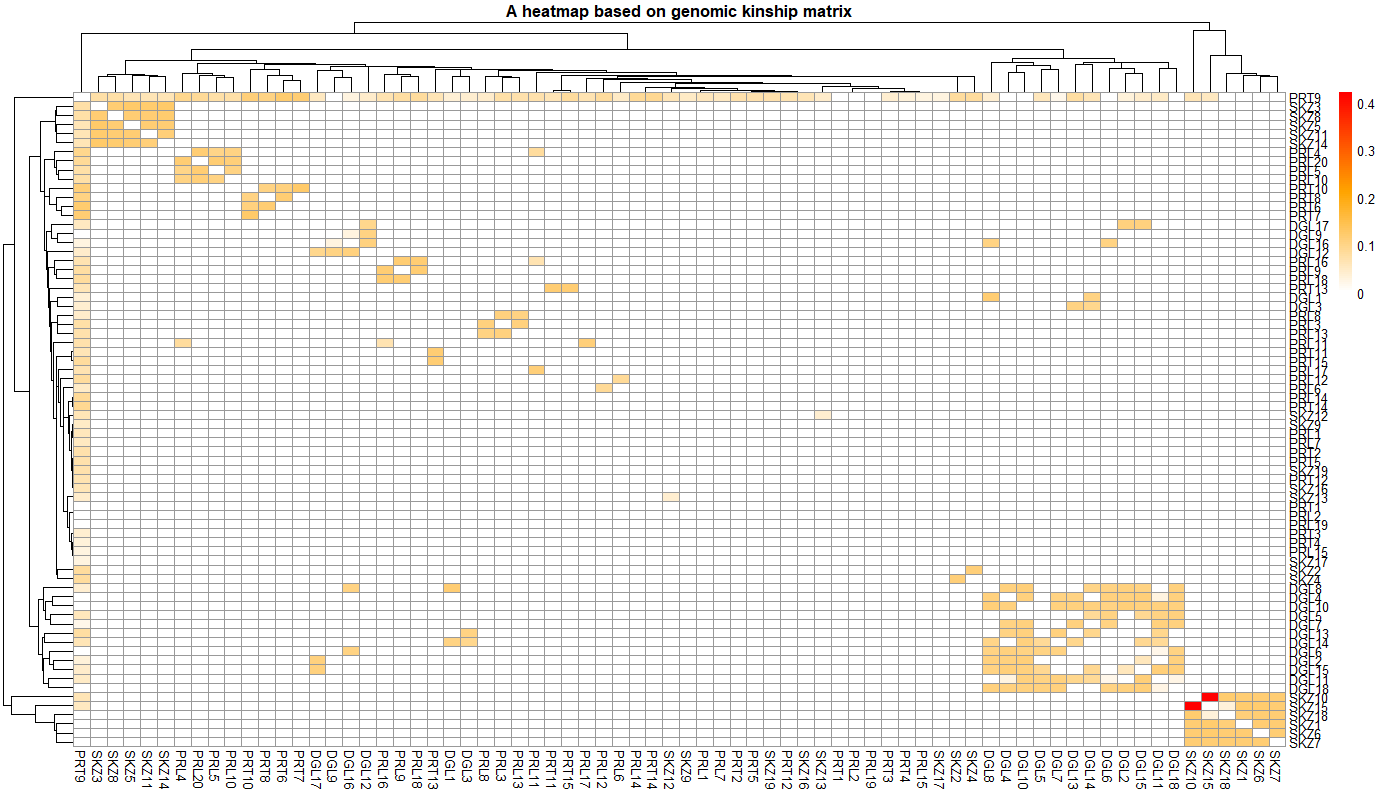


**Figure S1.** Genomic kinship matrix based on pairwise PI_HAT values. A single pair of individuals (SKZ10 and SKZ15) shows elevated relatedness, while all remaining individuals exhibit negligible genomic kinship.
